# Neural mechanisms underlying psilocybin’s therapeutic potential – the need for preclinical in vivo electrophysiology

**DOI:** 10.1177/02698811221092508

**Published:** 2022-05-30

**Authors:** Rebecca Smausz, Joanna Neill, John Gigg

**Affiliations:** 1 Division of Neuroscience and Experimental Psychology, Faculty of Biology, Medicine and Health, The University of Manchester, Manchester, UK; 2 Division of Pharmacy and Optometry, Faculty of Biology, Medicine and Health, The University of Manchester, Manchester, UK; 3 Medical Psychedelics Working Group, Drug Science, UK

**Keywords:** Gamma rhythm, neural network, parvalbumin, default-mode network, neuroplasticity, psychedelic medicine, 5HT2a receptor, psilocybin

## Abstract

Psilocybin is a naturally occurring psychedelic compound with profound perception-, emotion- and cognition-altering properties and great potential for treating brain disorders. However, the neural mechanisms mediating its effects require in-depth investigation as there is still much to learn about how psychedelic drugs produce their profound and long-lasting effects. In this review, we outline the current understanding of the neurophysiology of psilocybin’s psychoactive properties, highlighting the need for additional preclinical studies to determine its effect on neural network dynamics. We first describe how psilocybin’s effect on brain regions associated with the default-mode network (DMN), particularly the prefrontal cortex and hippocampus, likely plays a key role in mediating its consciousness-altering properties. We then outline the specific receptor and cell types involved and discuss contradictory evidence from neuroimaging studies regarding psilocybin’s net effect on activity within these regions. We go on to argue that in vivo electrophysiology is ideally suited to provide a more holistic, neural network analysis approach to understand psilocybin’s mode of action. Thus, we integrate information about the neural bases for oscillatory activity generation with the accumulating evidence about psychedelic drug effects on neural synchrony within DMN-associated areas. This approach will help to generate important questions for future preclinical and clinical studies. Answers to these questions are vital for determining the neural mechanisms mediating psilocybin’s psychotherapeutic potential, which promises to improve outcomes for patients with severe depression and other difficulty to treat conditions.

## Introduction

The profound cognition-, perception- and emotion-altering properties of psychedelic compounds have been recognized for centuries if not millennia. A prominent example is psilocybin, which was referred to as ‘teonanacatl’ or ‘God’s flesh’ by the Aztecs, who used it during their spiritual rituals (Nichols, 2020). It is naturally produced by over 200 species of basidiomycetes, the ‘sacred mushrooms’ of America, identified as part of the Psilocybe genus by mycologist Roger Heim in 1957 (Nichols, 2020). Rigorous scientific research into its properties only began after Heim sent a sample to Albert Hofmann, the Sandoz chemist who previously synthesized and characterized the psychoactive effects of the synthetic psychedelic compound lysergic acid diethylamide (LSD) (Hofmann, 1980). Similarly to LSD, this mushroom extract did not seem to alter mouse or dog behaviour but had a dramatic psychoactive effect when Hofmann ingested it himself. Thus, he went on to isolate its active compound and named it psilocybin (Hofmann et al., 1958). Great interest in uncovering psilocybin’s properties, mechanisms of action and potential therapeutic applications followed, leading to more than 100 published research articles by 1980 (Nichols, 2020). However, the rise in illicit drug use during the 1960s led to the ‘Controlled Substances Act’ under president Nixon, and similar action was taken by the United Nations (Nutt, 2015), placing psychedelics into the most tightly regulated drug category, Schedule I: substances with high abuse and no therapeutic potential (Belouin and Henningfield, 2018). This change limited funding and research possibilities for years; however, since the 1990s, efforts have re-emerged to promote psychedelic research, with small clinical trials demonstrating that they have great therapeutic potential (Nichols, 2020).

Psilocybin’s psychoactive effects were characterized by Griffiths et al., 2006, who found that it induced acute perceptual changes, such as distortions and pseudo-hallucinations, a sense of ego dissolution and union with the world and a wide spectrum of emotions, ranging from panic to euphoria, which peaked at 2 h and lasted for about 6 h after ingesting 30 mg of psilocybin. These effects fit into the three categories of Dittrich’s ‘Abnormal Mental States’ (APZ) questionnaire, which assesses altered states of consciousness, namely ‘Visionary Restructuralization’, ‘Dread of Ego Dissolution’ and ‘Oceanic Boundlessness’ (Dittrich, 1998) and, thus, somewhat resemble a psychotic episode in schizophrenia (Vollenweider et al., 1998). In addition, the acute psychedelic experience led to self-reported positive changes in attitudes and social interactions even 2 months after psilocybin administration (Griffiths et al., 2006), indicating that it might have long-lasting therapeutic benefits. Indeed, there is evidence that psilocybin can ameliorate symptoms of obsessive-compulsive disorder (Moreno et al., 2006), can treat both smoking (Johnson et al., 2014) and alcohol dependence (Bogenschutz et al., 2015) and, perhaps most promisingly, recent trial data show that it has great potential for treating severe depression (see review by Gonda et al., 2021). Indeed, several studies show that psilocybin can relieve end-of-life anxiety and depression-associated symptoms in patients with terminal cancer (Griffiths et al., 2016; Grob et al., 2011). Moreover, Carhart-Harris et al., 2016 found that administration of two oral doses (10 and 25 mg 7 days apart) of psilocybin improved symptoms in patients with treatment-resistant depression 1 week, 3 months and even 6 months later (Carhart-Harris et al., 2018). This suggests that psilocybin might be more effective for severe depression than current antidepressants, which require daily administration, have a significant side effect burden and are ineffective for many patients (Nutt et al., 2020). Thus, the Food and Drug Administration (FDA) granted ‘breakthrough therapy’ status for psilocybin for treatment-resistant depression and major depressive disorder (MDD) (Nichols, 2020). In fact, recent Phase-2 clinical trials comparing the antidepressant efficacy of two 25 mg oral doses of psilocybin versus escitalopram, a selective serotonin reuptake inhibitor presently used for treating MDD, found that both compounds successfully reduced self-reporting scores on the 16-item Quick Inventory of Depressive Symptomatology (QUID-SR-16) scale 6 weeks later, but importantly that secondary outcomes favoured psilocybin (Carhart-Harris et al., 2021). Also, psilocybin had fewer side effects and only required the administration of two doses instead of daily escitalopram treatment while maintaining its antidepressant benefits, which suggests that it might be preferable to current antidepressants for many patients.

It has been proposed that psilocybin’s therapeutic potential may result from resetting the brain’s connectivity patterns by opening a therapeutic window to facilitate the emergence of novel insights, potentially leading to emotional release (Carhart-Harris and Nutt, 2017). As such, psilocybin could potentially treat the causes of psychiatric illness and enable recovery, instead of only ameliorating symptoms as per current antidepressants (Nutt et al., 2020). However, the underlying neural mechanisms by which psilocybin produces such profound effects require further exploration. Therefore, in this review, we aim to summarize advances in the field by first describing how psilocybin affects brain regions associated with the default-mode network (DMN), especially the prefrontal cortex (PFC) and hippocampus (HC), which may explain its consciousness-altering properties. We then outline the specific cell and receptor types on which psilocybin acts, followed by discussing whether this leads to an increase or decrease in overall activity within these regions. Finally, we argue that investigating psilocybin-induced changes in neural oscillatory activity is one of the best ways to evaluate its effect on network activity within and across DMN-associated brain regions, stressing the need for more preclinical studies.

## Psilocybin and the DMN

Accumulating evidence indicates that the brain has an intrinsic network that is highly active during task-free, resting states (Buckner et al., 2008). During such passive states, a set of brain regions consistently show increased, temporally correlated spontaneous activity, indicating that they must be functionally connected and, thus, part of the same network (Greicius et al., 2003), which was termed the ‘DMN’ (Gusnard et al., 2001). Functional and anatomical imaging studies have identified the DMN as containing several cortical areas, including the PFC, a key mediator of executive function, including attention, multisensory integration and decision-making (Diamond, 2013), and the cingulate, parietal and temporal association cortices (Andrews-Hanna et al., 2010). In addition, more recent studies indicate that subcortical areas, such as the HC, the main structure involved in declarative memory processing (Eichenbaum, 2000), the amygdala, which is integral to emotion-related processes and the thalamus, the main sensory relay region, might also be associated with the DMN (Alves et al., 2019; Greicius et al., 2004). These findings have also been replicated in rodents, indicating that the DMN is a cross-species fundamental property of the brain (Lu et al., 2012).

**Figure 1.**
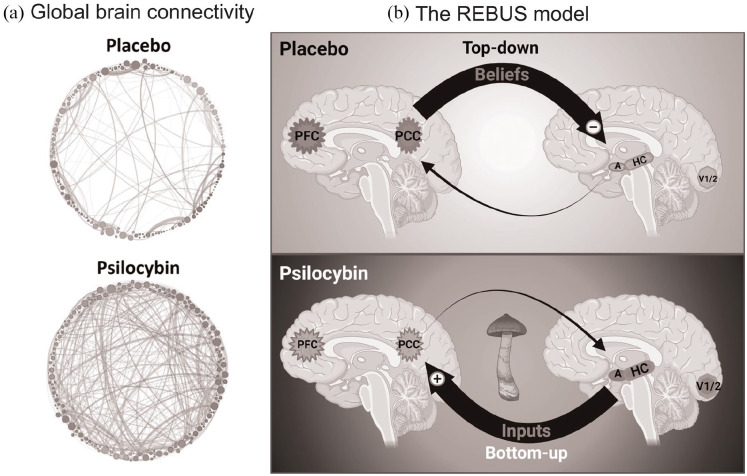
Psilocybin acts by altering the activity and connectivity across DMN-associated brain regions: (a) psilocybin increases and diversifies functional connectivity (=positively correlated neural activity) patterns throughout the brain. Nodes represent neuronal clusters, shades of grey delineate communities obtained by modularity, node size proportionate to degree of connectedness and edges are direct links between functionally connected areas. Figure was adapted with permission from Petri et al. (2014). (b) According to the ‘REBUS and the anarchic brain’ theory Carhart-Harris and Friston (2019), the top–down inhibitory control by which the PFC and PCC maintain prior beliefs (top) is decreased by psychedelics, enabling incoming inputs from HC, amygdala and sensory cortices to have a larger effect on the subsequent activation patterns (bottom). Thus, under normal circumstances, memory-, emotion- and sensation-related inputs have a more limited impact on the contents of consciousness. In contrast, psychedelics enhance bottom-up information flow by decreasing top–down inhibition, which leads to enriched experience, potentially enabling the emergence of novel insights with therapeutic benefits. PFC: prefrontal cortex; PCC: posterior cingulate cortex; HC: hippocampus; A: amygdala; V1/2: primary and secondary visual areas. Created with BioRender.com.

Psilocybin’s ability to induce altered brain states might be explained by its effect on the DMN. The abundance of connections within and between its core brain regions makes the DMN an ideal candidate for enabling the emergence of consciousness and self-referential processes (Buckner et al., 2008). As such, it has been hypothesized that psilocybin’s ability to induce altered states of consciousness is a result of it affecting information processing and integration across the DMN (Carhart-Harris et al., 2014). Thus, psilocybin is thought to increase brain entropy, a measure of the variety of accessible neural states, by causing a temporary reorganization of activation patterns and enabling the formation of new long-range connectivity patterns (Figure 1a) (Nutt et al., 2020; Petri et al., 2014), which can lead to more flexible cognition and emotional breakthroughs (Roseman et al., 2019). Furthermore, the PFC and the posterior cingulate cortex (PCC) play a key role in generating the concept of self (Brewer et al., 2013; Gusnard et al., 2001), so psilocybin’s effect of diminishing self–other discrimination, or even complete ego dissolution at higher doses (Vollenweider and Kometer, 2010), could be mediated through its action within these DMN-associated regions. Indeed, functional magnetic resonance imaging (fMRI) studies in humans found that psilocybin decreased activity within the PFC, PCC and other regions across the DMN, profoundly altering consciousness and self-perception (Carhart-Harris et al., 2012).

The PFC also integrates memory-related inputs from the HC (Sigurdsson and Duvarci, 2016) and stimuli with emotional valence from the amygdala (Lapate et al., 2016), using these to generate self-reflection (Jenkins and Mitchell, 2011), planning and decision-making to initiate goal-directed behaviour (Fuster, 2001). Activity within these regions is altered in depression, where excessive rumination and self-blaming are associated with PFC hyperactivity (Philippi et al., 2018), which receives input related to negative emotions from hyperactive amygdalae (Drevets et al., 2008) and disturbs self-referential memories from the HC (Godsil et al., 2013). Indeed, increased DMN activity has been found in depressed patients (Sheline et al., 2009) and animal models for depression (Alcaro et al., 2010). As such, disrupting the pathological activity patterns within and between DMN regions is a potential mechanism through which psilocybin may exert its long-lasting antidepressant effects (Figure 1(b)). This aligns well with the ‘relaxed beliefs under psychedelics (REBUS) and the anarchic brain’ hypothesis (Carhart-Harris and Friston, 2019), which claims that psychedelics decrease the influence of prior beliefs and expectations by decreasing top-down inhibition, thus, breaking the usual thinking patterns and enhancing bottom-up information transmission to enable the emergence of a new, potentially brighter perspective (Figure 1b).

However, whether psilocybin causes an overall decrease or increase in activity within DMN-associated regions is still a matter of debate and will be discussed in another section.

## Psilocybin – Receptors, cell types and brain regions

**Figure 2.**
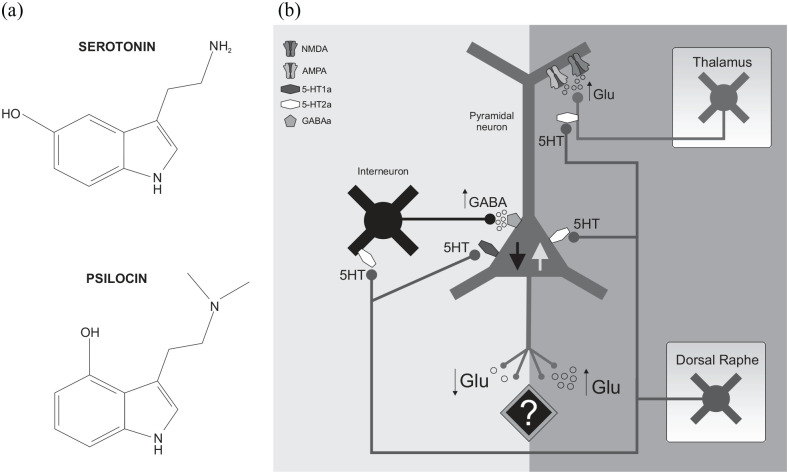
Effect of psilocin on receptors, cell types and PFC activity: (a) as the chemical structure of psilocin is very similar to that of serotonin (5HT), it can activate 5HT receptor subtypes and (b) psilocin may decrease pyramidal neuron (PYR) excitability (central downwards pointing black arrow) by stimulating GABA release from interneurons by 5HT2A-R activation (left) and by activating 5HT1A-R on PYR neurons. In contrast, psilocin can increase PYR activity (central upwards pointing grey arrow) by activating 5HT2A-R on PYR directly (projections arise from the dorsal raphe (DR) nucleus) and thalamo-cortical afferent terminals. This stimulates glutamate (Glu) release, which further depolarizes PYR neurons by opening their glutamatergic cation channels (AMPA and NMDA receptors).

Indoleamines are a class of plant-derived psychedelic compounds which include N, N-Dimethyltryptamine (DMT), 4-phosphoryloxy-DMT or psilocybin and 5-methoxy-DMT (5-MeO-DMT). Psilocybin is a pro-drug, which is metabolized by dephosphorylation to its active metabolite psilocin (4-hydroxy DMT) shortly after administration in rodents (Horita, 1962) and humans (Hasler et al., 1997) alike. Psilocin mediates all the downstream effects underlying psilocybin’s psychoactive properties (Nichols, 2004). Given the similarity between the chemical structures of psilocin and serotonin (5-hydroxy tryptamine – Figure 2a), an endogenous monoamine neurotransmitter involved in several cognitive functions, including flexible thinking, developing coping strategies in the face of adversity and mood regulation (Carhart-Harris and Nutt, 2017); it is not surprising that psilocin was also shown to act primarily by 5HT receptors (Halberstadt and Geyer, 2011). Although psilocin is a relatively non-selective 5HT receptor partial agonist, its psychoactive effects are most likely mediated by the 5HT2A receptor (5HT2A-R) subtype (Halberstadt and Geyer, 2011), as antagonizing these receptors with ketanserin prevents changes in perception, cognition and emotions following psilocybin administration in humans (Vollenweider et al., 1998). Moreover, the head-twitch response, the behavioural proxy for the psychedelic state in rodents, is completely blocked by 5HT2A-R antagonists (Halberstadt, 2015).

The 5HT2A-R is the predominant 5HT receptor subtype in the cortex and is highly expressed in DMN-associated structures, especially in the PFC and association cortices of both humans (Carhart-Harris and Nutt, 2017) and rodents (Amargós-Bosch et al., 2004), which play an important role in high-order cognitive processes, as discussed above. These receptors are G-protein-coupled receptors (GPCRs), which on activation cause an increase in host cell excitation through the Gq–phospholipase C signalling pathway (by IP3-induced Ca2 + release from intracellular stores) (Andrade, 2011). Interestingly, 5HT2A-Rs are present on both γ-aminobutyric-acid-ergic (GABA – the main inhibitory CNS neurotransmitter) interneurons (IN) and glutamatergic (glu – the main excitatory CNS neurotransmitter) PYR in the PFC (Andrade, 2011); moreover, 5HT2A-Rs are also present on excitatory thalamo-cortical afferents that synapse on Layer 5 PYR neurons in the PFC (Figure 2(b)) (Marek, 2017). As such, the question arises whether psilocybin, acting through its active metabolite psilocin, has an overall excitatory or inhibitory effect on neural activity within the PFC. On the one hand, through action as a partial agonist at 5HT2A-Rs, it can increase PYR neuron and thalamo-cortical afferent activity (Figure 2b), resulting in a large increase in PFC glutamate release, which might explain the sensory flooding and altered perception experienced by subjects following psilocybin administration (Vollenweider and Preller, 2020). On the other hand, it can also stimulate GABA release from INs (Figure 2b), which in turn inhibits PYR neurons and could, therefore, counteract any excitatory effects. Psilocin also acts as a partial agonist at the 5HT1A receptor (5HT1A-R) subtype), a Gi-GPCR inhibiting the activity of adenylate cyclase, leading to a decrease in host cell excitation (decreased PKA-mediated extracellular Ca2 + influx) (Halberstadt and Geyer, 2011). As most PFC PYR neurons co-express the 5HT2A-R and 5HT1A-R in humans (Carhart-Harris and Nutt, 2017) and rodents (Amargós-Bosch et al., 2004), while a quarter of the INs also express these (Carhart-Harris and Nutt, 2017; Santana et al., 2004), it is difficult to predict psilocin’s overall effect on activity within this region. This might explain why some studies detected an increase, while others a decrease in PFC activity following psilocybin administration, which is discussed in the next section.

In addition, it would also be important to determine the effect of psilocybin on hippocampal activity, given that the HC plays a key role in declarative (Eichenbaum, 2000) memory processes, including self-referential memories (Bartsch et al., 2011), and that a HC–PFC interplay orchestrates several high-level cognitive processes (Eichenbaum, 2017) that are affected by psychedelics (Mason et al., 2020). Moreover, the PFC and HC are two of the main regions affected by depression, in which they show marked atrophy (Drevets et al., 2008), so the antidepressant potential of psychedelics might also involve altering activity within the HC. The 5HT receptor subtype expression profiles support this hypothesis, as although there is a higher 5HT1A-R expression level in the HC on both PYR and INs of humans and rodents (Aznar et al., 2003; Carhart-Harris and Nutt, 2017), 5HT2A-Rs are also present on both cell types in the HC (Carhart-Harris and Nutt, 2017; Lüttgen et al., 2004). Moreover, 5HT1A-Rs are also involved in mediating effects of psychedelics, as for example Riga et al., 2018 showed that 5-MeO-DMT, which like psilocybin is a 5HT1A-R and 5HT2A-R partial agonist indoleamine psychedelic, retained some psychoactive effects in 5HT2A-R knockout mice, which were abolished by 5HT1A-R antagonists. Thus, psilocybin’s effect on HC activity is also worth investigating.

Indeed, stimulating neuroplasticity within the PFC and HC might be a potential mechanism underlying psychedelics’ antidepressant effects. For example, psychedelics accelerate activation of ionotropic glutamatergic AMPA (Zhang and Marek, 2008) and NMDA receptors (Barre et al., 2016), potentially by stimulating glutamate release from PYR and cortico-thalamic afferent neurons on 5HT2A-R activation (Figure 2(b)) (Vollenweider and Kometer, 2010). alpha-amino-3-hydroxyl-5-methyl-4-isoxazole-propionate (AMPA) and N-methyl-D-aspartate (NMDA) receptor stimulation leads to an increase in intracellular brain-derived neurotrophic factor (BDNF) (Vaidya et al., 1997), which increases synapse number and strength by stimulating long-term potentiation (Rex et al., 2007). Indeed, low doses (0.1 and 0.5 mg/kg) of psilocybin promoted neurogenesis in mouse HC (Catlow et al., 2013). In addition, 1 mg/kg psilocybin (corresponding to a dose of 10 mg in a 65 kg human when accounting for pharmacokinetic and allometric differences (Nair and Jacob, 2016) caused a 10-fold increase in spine density and size in the medial PFC (mPFC) of mice, which persisted for 7 days and, although slightly diminished, was still detectable 34 days after administration (Shao et al., 2021). These structural changes were accompanied by a reduction in escape failures in the learned helplessness paradigm, which evaluates stress-induced depressive symptomatology (Shao et al., 2021). These results match recent findings in pigs, where psilocybin increased presynaptic density in HC and mPFC both 1 and 7 days after administration (Raval et al., 2021). Moreover, other psychedelics, such as DMT and LSD, stimulated plasticity by increasing synaptic density, dendritic arborization and spinogenesis in rat mPFC both in vitro and in vivo, with LSD almost doubling the number of dendritic spines per unit length (Ly et al., 2018). These effects were mediated by the activation of the TrkB-mTOR pathway, as co-administration of the mTOR inhibitor rapamycin completely blocked psychedelics’ plasticity-promoting effects (Ly et al., 2018). Thus, rescuing the marked neuronal loss in the PFC and HC seen in depression (Holmes et al., 2019) is a possible mechanism underlying psilocybin’s psychotherapeutic potential, which will be very important to fully understand.

Another potential mechanism through which psychedelics might improve depression-associated symptoms is by normalizing 5HT2A-R density (Vollenweider and Kometer, 2010), which is pathologically elevated in MDD patients (Meyer et al., 2003; Shelton et al., 2009). For example, a single dose of psilocybin administration decreased 5HT2A-R levels in the mPFC and HC of pigs (Raval et al., 2021). In addition, chronic LSD administration normalized 5HT2A-R levels in rat PFC (Gresch et al., 2005), likely through a compensatory homeostatic downregulation following repeated overactivation by LSD. However, the psychedelic-induced 5HT2A-R downregulation was found to be only transient, lasting up to 48 h post-administration (Buckholtz et al., 1990; Raval et al., 2021). As 5HT2A-Rs play a key role in mediating anxiety-related behaviours (Weisstaub et al., 2006), their transient psychedelic-induced downregulation might have immediate effects on ameliorating depression-associated symptoms, such as reducing anxiety levels and improving subjects’ general sense of well-being (which is indeed often reported in the so-called ‘after glow’ period lasting for a couple of days following the psychedelic experience (Carhart-Harris and Nutt, 2017)). This might allow patients to maximize the benefits of the window of increased flexibility to reprocess emotions and self-referential memories opened by psychedelics, potentially leading to a brighter perspective. Thus, it would be interesting to investigate whether psilocybin does indeed transiently decrease 5HT2A-R levels in human subjects as well, and if so, whether the extent of this decrease might correlate with subsequent improvements in depression-associated symptoms.

## Psilocybin – Increase or decrease in activity within DMN-associated regions?

Given the heterogeneity of psilocybin’s cell and receptor type targets, it is still a matter of debate whether it causes an overall increase or decrease in activity across DMN-associated brain regions. The main advocates of the hypothesis that psilocybin increases PFC activity are a Zürich-based group (Vollenweider and Kometer, 2010; Vollenweider and Preller, 2020). They were the first to investigate psilocybin’s effect on human brain activity and found a global increase in glucose metabolism with positron emission tomography (PET), which is indicative of neuronal activation and was most prominent in the PFC, anterior cingulate and temporomedial cortex (Vollenweider et al., 1997). This increase also positively correlated with experiencing ego dissolution and perceptual changes, so the authors concluded that psilocybin induces hyperfrontality (i.e. PFC hyperactivity), resembling an acute episode of psychosis in schizophrenia. They also demonstrated that these effects are mediated by psilocin acting as an agonist at 5HT2A-R (Vollenweider et al., 1998), which induces the activation of glutamatergic Layer 5 PYR neurons and thalamo-cortical afferents, as described in the previous section (Figure 2b). More recent studies suggest that, in addition to Layer 5 PYR neurons, which co-express 5HT2A-R and 5HT1A-R, there is an additional subset of Layer 6 PYR neurons which express the excitatory 5HT2A-R only (Andrade, 2011). On activation by psilocin, these should release a large amount of glutamate, which could spread the activation within the PFC (Béïque et al., 2007). Indeed, a magnetic resonance spectroscopy imaging study detected an increase in PFC glutamate levels following psilocybin administration in humans (Mason et al., 2020). Furthermore, in vivo microdialysis studies evaluating the effect of LSD on rat PFC found that it caused a marked increase in extracellular glutamate concentration, which was blocked by 5HT2A-R antagonists (Muschamp et al., 2004). Moreover, brain slice electrophysiological recordings from rat PFC Layer 5 PYR neurons detected an increase in excitatory postsynaptic currents on administration of the 5HT2A/C-R agonist psychedelic compound 2,5-Dimethoxy-4-iodoamphetamine (DOI), which was abolished by AMPA receptor antagonists (Zhang and Marek, 2008). All these studies support the hypothesis that psychedelics increase glutamatergic signalling within the PFC, leading to an increase in overall network activity.

As these findings build a strong case to support the hypothesis that psilocybin causes an overall increase in activity within DMN-associated regions, it came as a surprise that a London-based group could only detect deactivation and decoupling throughout the DMN following psilocybin administration in a human fMRI study (Carhart-Harris et al., 2012). These included a decrease in functional connectivity between the PFC and PCC, which correlated with psilocybin’s perception- and cognition-altering effects. They argued that their imaging method, fMRI, is more suitable for evaluating such changes than PET, which the Swiss group used, given fMRI’s superiority in terms of temporal resolution (Carhart-Harris et al., 2012). They also highlighted psilocybin’s ability to excite INs using the 5HT2A-R, thus causing a marked increase in PFC GABA release (Figure 2b), which has an inhibitory effect on PYR neuron activity (Andrade, 2011), as discussed in the previous section. Indeed, Abi-Saab et al., 1999 found a marked increase in extracellular GABA levels in rat PFC following DOI administration; however, they also detected elevated levels of the neuronal activity marker c-fos in both INs and PYR neurons, indicating that DOI activated both excitatory and inhibitory PFC neurons. Thus, it is also possible that the increase in GABA release was a compensatory mechanism to balance the increase in PYR activity. Another study showed that administration of 5HT directly into mouse PFC increased the firing rate of a subset of INs, namely the fast-spiking, parvalbumin-containing INs, by activating 5HT2A-Rs (Athilingam et al., 2017). However, although these studies show that 5HT2A-R agonists, including psilocin, activate inhibitory INs, this does not necessarily imply that the subsequent inhibition is sufficient to counter the concomitant increase in excitatory PYR neuronal activity.

An additional argument for the inhibitory theory comes from the studies highlighting the importance of 5HT1A-R-induced inhibition. Thus, when the DR nucleus, the main serotonergic source of the mammalian brain (Figure 2(b)), was electrically activated, the subsequent increase in 5HT was found to inhibit 60% of recorded rat PFC neurons (Hajós et al., 2003). This was mediated by 5HT1A-R activation, as effects were blocked by 5HT1A-R, but not 5HT2A-R antagonists (Hajós et al., 2003). Another study obtained very similar results, namely, DR activation inhibited the activity of 66% of studied rat PFC neurons, while also showing that this was mediated not only by 5HT1A-R but also by GABA_A_ receptor agonism, bringing further evidence for IN involvement (Puig et al., 2005). However, psychedelics have different 5HT receptor subtype affinity profiles compared to 5HT, as for example, psilocybin has higher affinity for 5HT2A-R over 5HT1A-R (see Halberstadt and Geyer, 2011) for rodent data, and (Neill et al., in press) for a thorough critical evaluation of human data, while the opposite is true for 5HT (Carhart-Harris and Nutt, 2017). Thus, psilocybin and other psychedelics with higher 5HT2A-R affinity might have the opposite effect on PFC activity compared to 5HT, which requires further investigation.

As such, there is evidence to support both theories, however, evaluating neurotransmitter levels and individual cell activity is still not sufficient for determining psilocybin’s effect on overall network activity within DMN-associated regions. Although functional imaging studies attempted to address this question, they can only measure neuronal activity indirectly through metabolic markers, such as blood volume, oxygen or glucose level changes (Logothetis, 2008). Moreover, different imaging technologies and data analyses have led to opposing findings. For example, the Swiss group argues that the psilocybin-induced decrease in DMN activity seen by the British group (Carhart-Harris et al., 2012) resulted from the latter’s failure to adjust for global brain perfusion levels, which is a necessary step because 5HT2A-R agonists have a confounding haemodynamic effect by decreasing vascular tone (Lewis et al., 2017). Thus, they repeated the fMRI study and found that, although psilocybin induced a global decrease in activity when measuring absolute cerebral blood volume changes, it caused a hyperfrontal activation pattern when measuring relative volume changes after baseline subtraction (Lewis et al., 2017), supporting the argument that psilocybin acts by increasing PFC activity. However, given the heterogeneity of the functional imaging results, a direct measure of neuronal network activity, such as in vivo electrophysiology, has greater potential to disentangle the overall effect of psilocybin on activity within and between the PFC and other DMN-associated regions.

## Local field potentials and spectral properties of neural oscillatory activity

In vivo electrophysiology is a high spatiotemporal resolution imaging method that can be used to record local field potentials (LFP), which are extracellular electric potentials that largely reflect the summed synaptic activity of neighbouring cells (within ~250 μm radius from the recording electrode) (Wang, 2010), particularly postsynaptic potentials in neuronal dendrites (Monosov et al., 2008). As such, LFPs can convey changes in spontaneous oscillatory activity, an intrinsic property of the mammalian brain arising from synchronized activity of populations of neurons (Bartos et al., 2007), which is indicative of neural network integrity (Buzsáki and Draguhn, 2004). The properties of these oscillations can be evaluated from the power spectrum of the recorded signal, covering a wide range of frequencies (0.5–200 Hz) and amplitudes, depending on the state of consciousness, the task being performed and the specific brain regions from which they are recorded (Buzsáki and Draguhn, 2004). Thus, high-frequency, low-amplitude oscillations, such as gamma waves (30–90 Hz) are associated with alertness and intense cognitive activity (Steriade, 1996) while, at the opposite end of the spectrum, the low-frequency, high-amplitude waves, also called slow waves, are characteristic of rest (alpha: 8–12 Hz; theta: 3–8 Hz) and sleep (delta: 0.5–3 Hz) (Harmony, 2013; McCormick and Bal, 1997). Oscillatory activity has been detected throughout the DMN and there is accumulating evidence that synchronization of activity in a specific power band is important for information processing within and across DMN-associated regions, particularly in the PFC and HC (Engel et al., 2001; Fries, 2015). As such, evaluating the psilocybin-induced changes in spectral power has great potential for determining its overall effect on activity within DMN-associated regions.

**Figure 3.**
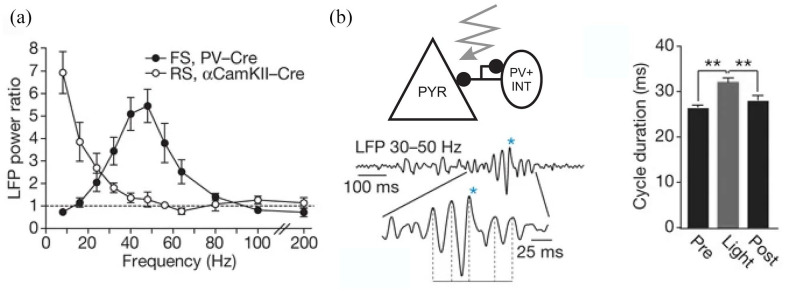
Parvalbuminergic interneurons are the main drivers of gamma oscillatory activity: (a) frequency distribution of oscillatory power induced by stimulation of parvalbumin-positive interneurons (PV INT), but not PYR neurons, shows an increase in gamma power and (b) duration of spontaneously occurring gamma waves is prolonged by optogenetic stimulation of PV INs with blue light (crooked arrow; **p < 0.01). Adapted with permission from Cardin et al. (2009). LFP: local field potentials; FS: fast-spiking; RS: regularly spiking; PYR: pyramidal neuron.

Both groups mentioned in the previous section agree that psychedelics induce an increase in neural signal diversity as measured by the Lempel–Ziv complexity level, which quantifies the informational content of an experience (Schartner et al., 2017; Vollenweider and Preller, 2020), by desynchronizing activity between associative hubs within the DMN (Muthukumaraswamy et al., 2013). This suggests that psychedelics should increase activity in the gamma frequency band, which is associated with high-level cognitive functions (Misselhorn et al., 2019) altered during the psychedelic experience (Kometer et al., 2015). Indeed, the neurophysiology of gamma rhythm generation and psilocin’s receptor targets suggest that psilocybin might induce increased gamma synchrony throughout the DMN. There are two main network models that are thought to generate gamma rhythms: inhibitory–inhibitory (I-I) and excitatory–inhibitory (E-I) loops, which consist of alternating activity of interconnected neurons (Wang, 2010). Accumulating evidence suggests that the inhibitory component of both of these is provided by GABA-ergic IN activity, which can generate gamma oscillations either by mutual inhibition (I-I) or entrain rhythmic activity in large populations of PYR neurons by feedback inhibition (E-I model) (Cardin et al., 2009; Wang, 2010). For example, Whittington et al., 1995 showed that the gamma oscillations induced by metabotropic glutamatergic receptor agonists in rat HC slices were blocked by the GABA_A_ receptor antagonist bicuculline, but not AMPA receptor antagonists. This indicated that inhibitory IN activity is necessary and might be sufficient for generating gamma oscillations, lending support to the I-I model. However, studies have shown that an interplay between inhibitory INs and excitatory PYR neurons (E-I model) can also generate gamma oscillations, although INs were the main drivers. For example, Sohal et al. (2009) showed that optogenetic excitation of a subset of INs in the mouse PFC, namely the fast-spiking parvalbumin (PV)-positive INs, markedly increased gamma oscillation power, as opposed to lower frequency bands. This finding was replicated by Cardin et al., 2009 in the mouse barrel cortex, where optogenetic stimulation of PV INs, but not PYR neurons, increased the power (Figure 3a) and duration of spontaneously occurring gamma oscillations (Figure 3b). However, PYR neurons were also found to play a key role where, for example, stimulating them in the mouse HC led to increased gamma power by driving excitation of basket cells, which are also PV INs (Alexander et al., 2009). Moreover, another study found that selective optogenetic pulsed-light stimulation of PYR neurons in cat visual areas led to a robust increase in gamma power (Ni et al., 2017). These studies suggest that reciprocally connected PYR neurons and INs, particularly the fast-spiking and PV-positive subtype, are involved in generating rapidly alternating patterns of inhibition and excitation as part of gamma rhythms. Therefore, as psilocybin can stimulate the activity of both PYR and IN cell types by 5HT2A-R activation, it is likely that it might also lead to an increase in gamma oscillatory activity and thus information processing within DMN-associated regions, a hypothesis worth testing.

## Psychedelic drug effect on neural oscillations – The need for more preclinical studies

### Increase in high-frequency electroencephalography power

**Figure 4.**
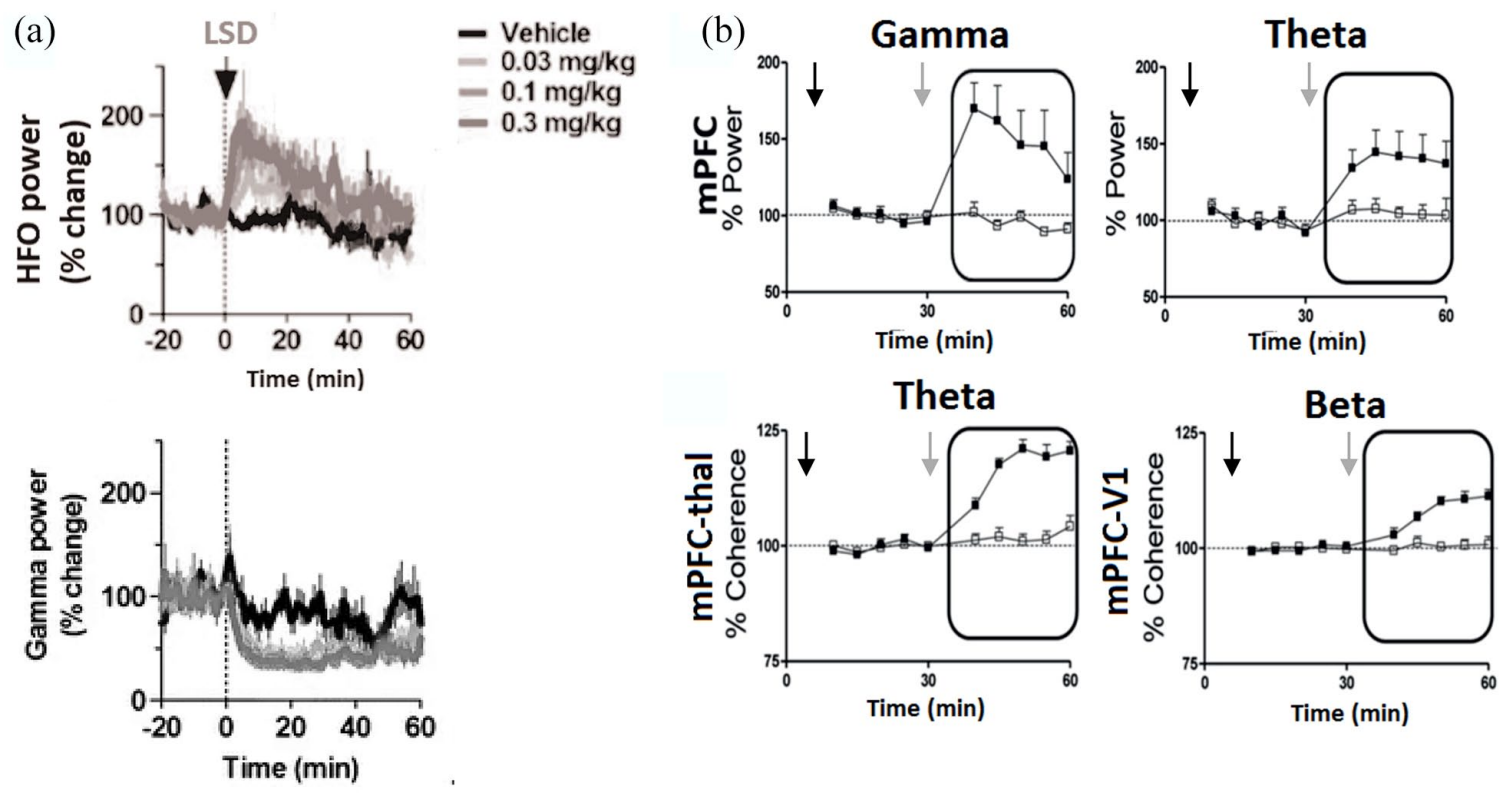
Effects of psychedelics on oscillatory activity within the rodent brain: (a) time-course shows a dose-dependent increase in 130–180 Hz high-frequency oscillations (HFO) and decrease in low gamma power in rat nucleus accumbens following LSD administration (black arrow). Adapted with permission from (Goda et al., 2013). (b) Time-courses show that 5-MeO-DMT increased mPFC gamma and theta power in freely moving mice (top row). 5-MeO-DMT increased coherence for mPFC–thalamus theta and mPFC–primary visual cortex beta rhythms in freely moving mice. Grey arrows: 5-MeO-DMT administration, black arrows: saline injection. Adapted with permission from Riga et al. (2018).

Psilocybin was shown to increase gamma power in a human quantitative electroencephalography (EEG) study (Tyls et al., 2016), although this was in contradiction with a previous magnetoencephalography (MEG) study that could only find widespread gamma power decrease throughout the DMN (Muthukumaraswamy et al., 2013). However, evidence is still sparse regarding psilocybin modulation of gamma in human studies, and there are very few preclinical studies that investigated the mechanisms involved in the mode of action of psychedelics. Indeed, this is one of the few fields in which animal work lags human studies (Wood et al., 2012). To our knowledge, the only rodent study to date that evaluated the effect of psilocybin on neural oscillatory activity is a recent one in mice, which found that 2 mg/kg psilocin induced a slight (1.2-fold) increase in mPFC high gamma (> 60 Hz) power during normal wakefulness and a 1.3-fold increase in awake, sleep-deprived mice (Thomas et al., 2021). There is also some preclinical evidence with other psychedelic compounds to support the hypothesis that they would increase gamma power in human brain (Figure 4). For example, the indoleamine 5-MeO-DMT was found to significantly increase gamma (30–80 Hz) and theta (4–10 Hz) power within the mPFC of freely moving mice (Figure 4b – (Riga et al., 2018)). In addition, the oneirogenic atypical psychedelic compound ibogaine induced a marked increase in high gamma (30–100 Hz) power in awake rats, consistent across sensory and motor cortices, and the olfactory bulb (González et al., 2021). Although even fewer studies evaluated psychedelics’ effect on HC activity, electrophysiological recordings from rat CA3 slices revealed that the 5HT2A/C-R agonist DOI prevented the decrease in carbachol-induced gamma (20–40 Hz) power seen after 5HT treatment (Krause and Jia, 2005). These findings indicate that psychedelics would indeed increase gamma power within DMN-associated regions.

However, other studies suggest that this effect might be region- and 5HT receptor subtype affinity-specific. For example, a study in awake rats showed that while the 5HT2A/C-R partial agonists LSD and DOI dose-dependently increased the power of very-high-frequency (130–180 Hz) oscillations in the nucleus accumbens, there was no effect on high gamma (70–90 Hz) and low gamma power (30–70 Hz) decreased (Figure 4(a) – (Goda et al., 2013)). Moreover, DOI was found to decrease gamma power (30–80 Hz) in freely moving rats’ anterior cingulate and orbitofrontal cortices (Wood et al., 2012), the former being part of the rodent PFC equivalent (Laubach et al., 2018). Also, increasing 5HT levels in rat mPFC through electrically stimulating the DR nucleus was found to decrease gamma power (Puig et al., 2005). This discrepancy between different psychedelics’ effects on gamma oscillatory activity is likely to be caused by differences in their 5HT receptor subtype affinity profiles (a comprehensive Ki database available as part of NIMH’s Psychoactive Drug Screening Programme at https://pdsp.unc.edu/databases/kidb.php), and brain-region-specific cytoarchitectural and functional properties. Therefore, the region-specific effect of each compound on oscillatory activity needs to be determined.

A psychedelic-induced increase in gamma power aligns well with the fact that gamma is strongest during complex mental tasks involving increased information processing, such as memory encoding and recall mediated by the HC (Bartos et al., 2007; Düzel et al., 2010), and performing attention and multisensory integration-demanding tasks to compute the optimal course of action by the PFC (Benchenane et al., 2011; Misselhorn et al., 2019), all of which are altered following psychedelic administration (Kometer et al., 2015). Increasing high gamma power also fits well with insights from human imaging studies that used graph theory and dynamic functional connectivity to model the effect of psychedelics on neural network properties. For example, using resting-state fMRI data, Luppi et al., 2021 found that LSD diversified functional connectivity patterns by increasing their divergence from structural constraints. Thus, LSD increased the neural networks’ small-world propensity by increasing their clustering coefficient (i.e. the degree of connectedness of neighbouring regions) and decreasing the path length (i.e. how quickly the information from one highly interconnected cluster of brain regions can reach another anatomically distant one), resulting in higher functional complexity (Luppi et al., 2021). Thus, psychedelics appear to have the opposite effect to general anaesthesia-induced loss of consciousness, in which the neural networks’ small-world character is diminished (Luppi et al., 2019). This is also in agreement with previous findings that psilocybin and LSD increased neural signal diversity as measured by the Lempel–Ziv complexity level, which quantifies the informational content of an experience and is much higher during waking consciousness, when gamma activity is strongest, compared to sleep or anaesthesia (Schartner et al., 2017). Thus, increasing gamma power is a plausible mechanism involved in mediating psychedelics’ psychoactive properties, however, the region- and compound-specific differences require further investigation.

### Decrease in low-frequency EEG power

There is also converging evidence from human and animal studies that psychedelics decrease the power of low-frequency oscillations, particularly in the mPFC. Thus, 5-MeO-DMT was found to cause a 31% decrease in delta (<4 Hz) oscillations in the mPFC of anaesthetized rats (Riga et al., 2014), which was very similar to the previously reported effect of DOI on anaesthetized rats’ mPFC (Celada et al., 2008). This mechanism of action is also supported by a human MEG study that found psilocybin decreased low-frequency oscillatory power, particularly within DMN posterior association cortices (Muthukumaraswamy et al., 2013). Interestingly, both DOI and 5-MeO-DMT–induced reductions in low-frequency oscillatory power were reversed by the antipsychotics clozapine and haloperidol, which act as 5HT2A-R and dopaminergic D2 receptor antagonists, respectively, while 5-MeO-DMT’s effects were also blocked by metabotropic glutamate mGlu2/3 receptor agonists (Celada et al., 2008; Riga et al., 2014). As the non-competitive NMDA receptor antagonist phencyclidine, which is known to induce schizophrenia-associated symptoms in humans and preclinical models, also decreases low-frequency oscillatory power, which is rescued by antipsychotics (Kargieman et al., 2007), these findings further support the hypothesis that psychedelics share common neural mechanisms with psychoses (Vollenweider et al., 1998). Therefore, insights from schizophrenia research might be useful for guiding future investigations into psychedelics’ effects on neural oscillations, particularly for low-frequency bands.

A psychedelic-induced decrease in delta power also fits well with Carhart-Harris et al.’s REBUS model (Carhart-Harris and Friston, 2019). This is because delta rhythms are now recognized to be more than just a marker of minimal neural activity during slow-wave sleep; similarly to alpha waves’ role in humans, delta oscillations were found to be involved in inhibiting task irrelevant information during concentration-demanding tasks (Harmony, 2013), and inhibiting emotional and reward-seeking drives (Knyazev, 2007). Therefore, by decreasing delta power, psychedelics might diminish the top-down inhibitory control of the PFC over subcortical areas, such as the HC, amygdala and sensory areas, thus, enabling increased memory-, emotion- and sensation-related information transmission and processing (Figure 1b). This might be a potential mechanism underlying psychedelics’ ability to open a ‘therapeutic window’, as postulated by Carhart-Harris and Friston, 2019, in which the resulting more flexible thinking patterns could lead to the emergence of a novel perspective with potential psychotherapeutic benefits. This may be particularly important for depression and anxiety-related disorders, in which psychedelics could break the PFC-centred rumination and negative thinking patterns and facilitate overcoming past traumas. Therefore, the effect of psychedelics on delta oscillations within DMN-associated regions deserves further investigation.

### Effect on cross-regional coherence

In addition to evaluating psychedelic drug effects on individual brain regions, determining how they affect network oscillatory activity across different areas is also of great interest and value. Coherence is a measure of how well oscillatory activity in one region temporally correlates with that in another, presumably by long-range communication within networks that support cognitive processes (Fries et al., 2015). Although few studies have evaluated psychedelics’ effect on cross-regional coherence, Riga et al., 2018 found an increase in theta and beta band coherence between mPFC and the medial dorsal thalamic nucleus, and enhanced beta coherence between mPFC and the primary visual cortex following 5-MeO-DMT administration in freely moving mice (Figure 4b). However, little is known about how psychedelics may alter mPFC–HC coherence, and studies evaluating the effect of 5HT on this are also lacking (Puig and Gener, 2015). This is of major interest because mPFC–HC coherence is crucial for many aspects of cognition; for example, the HC theta rhythm entrains gamma oscillatory activity within the mPFC as part of high-level cognitive tasks involving multisensory integration and memory-related processes (Eichenbaum, 2017; Siapas et al., 2005), which are altered by psychedelics (Kometer et al., 2015). Therefore, more preclinical in vivo electrophysiology studies would be particularly useful to determine the effect of psychedelics on cross-regional coherence, particularly between the PFC and HC, but also other regions which play a key role in the psychedelic experience, such as the PCC, amygdala and sensory areas (Figure 1b), among others.

## Conclusion

Despite the setbacks that hindered progress in psychedelic research following legal restrictions, in the last three decades, there has been an increase in the number of studies investigating their mode of action and therapeutic applications. Of these, psilocybin has shown great potential for treating psychiatric disorders, especially severe depression and anxiety-related conditions. This is thought to be due to its ability to induce altered states of consciousness, which might then facilitate the emergence of a novel perspective, leading to emotional release, as postulated by the REBUS model. Evidence suggests that psilocybin’s effects are primarily mediated by activation of the 5HT2A-R and 5HT1A-R within DMN-associated brain regions, particularly the PFC and HC, which play key roles in sustaining consciousness- and self-related cognitive processes. However, psilocybin’s overall effect on neuronal activity within these regions is still unclear, given that it can directly excite inhibitory INs, excitatory PYR neurons and thalamo-cortical afferents by activating 5HT2A-Rs, while it can also lower IN and PYR neuron excitability by activating 5HT1A-Rs. Functional imaging studies attempted to address this question, but different groups reached opposite conclusions. These, however, relied on indirect measures of neuronal activation, so using a technique with higher spatiotemporal resolution to measure excitable-cell generated activity directly, such as in vivo electrophysiology, is likely to be better suited for evaluating psilocybin’s overall effect within and across DMN-associated brain regions. A handful of studies suggest that psychedelics act by increasing oscillatory power in high-frequency EEG bands, which fits well with their ability to excite both PYR and INs, the alternating activation of which is involved in gamma rhythm generation within the mammalian PFC and HC. A few other studies also found that psychedelics decreased low-frequency oscillatory power within the PFC and increased theta and beta band coherence between the PFC and sensory areas. However, evidence is still sparse; for example, in the case of psilocybin, a single preclinical study and a couple of human EEG and MEG studies to date evaluated its effect on mPFC neural oscillatory activity, with no data on its effect within the HC, amygdala or sensory areas, nor on cross-regional coherence between DMN-associated regions. Therefore, there is an urgent need for additional preclinical studies to address these questions and determine the mechanisms underlying psilocybin’s psychotherapeutic potential, which could prove highly informative for designing future clinical studies and novel molecules and is most likely to lead to the development of improved treatments for hard-to-treat psychiatric and neurological disorders.
